# Nanotoxicity: An Interplay of Oxidative Stress, Inflammation and Cell Death

**DOI:** 10.3390/nano5031163

**Published:** 2015-06-30

**Authors:** Puja Khanna, Cynthia Ong, Boon Huat Bay, Gyeong Hun Baeg

**Affiliations:** Department of Anatomy, Yong Loo Lin School of Medicine, National University of Singapore, 4 Medical Drive, MD10, Singapore 117 597, Singapore; E-Mails: puja_khanna@u.nus.edu (P.K.); cynthiaong@u.nus.edu (C.O.); boon_huat_bay@nuhs.edu.sg (B.H.B.)

**Keywords:** nanoparticles, nanotoxicity, ROS generation, oxidative stress, inflammation, DNA damage, apoptosis

## Abstract

Nanoparticles are emerging as a useful tool for a wide variety of biomedical, consumer and instrumental applications that include drug delivery systems, biosensors and environmental sensors. In particular, nanoparticles have been shown to offer greater specificity with enhanced bioavailability and less detrimental side effects as compared to the existing conventional therapies in nanomedicine. Hence, bionanotechnology has been receiving immense attention in recent years. However, despite the extensive use of nanoparticles today, there is still a limited understanding of nanoparticle-mediated toxicity. Both *in vivo* and *in vitro* studies have shown that nanoparticles are closely associated with toxicity by increasing intracellular reactive oxygen species (ROS) levels and/or the levels of pro-inflammatory mediators. The homeostatic redox state of the host becomes disrupted upon ROS induction by nanoparticles. Nanoparticles are also known to up-regulate the transcription of various pro-inflammatory genes, including tumor necrosis factor-α and IL (interleukins)-1, IL-6 and IL-8, by activating nuclear factor-kappa B (NF-κB) signaling. These sequential molecular and cellular events are known to cause oxidative stress, followed by severe cellular genotoxicity and then programmed cell death. However, the exact molecular mechanisms underlying nanotoxicity are not fully understood. This lack of knowledge is a significant impediment in the use of nanoparticles *in vivo*. In this review, we will provide an assessment of signaling pathways that are involved in the nanoparticle-induced oxidative stress and propose possible strategies to circumvent nanotoxicity.

## 1. Introduction

Nanotechnology encompasses the study and manipulation of particles at the nanoscale (1–100 nm) level, commonly known as nanoparticles [1]. Nanoparticles have unique mechanical and physicochemical properties due to their increased relative surface area and quantum effects, favoring their usage in various applications [2,3]. In the past decade, the field of nanotechnology has received considerable attention due to its wide variety of applications being extended to the biotechnology, electronics, aerospace and computer industry. More recently, nanotechnology is also applied to the field of nanomedicine, which covers nanotechnology-based diagnosis, treatment and prevention of human diseases such as cancer, improving human health and well-being [4].

Nanoparticles are frequently used as a tool for drug delivery in nanomedicine. They can be categorized into several different groups such as polymers, inorganic nanoparticles and metallic nanoparticles, depending on their physicochemical properties.

Polymers such as polysaccharide chitosan nanoparticles (CS-NPs) function in drug delivery due to their ability to facilitate both protein and drug conjugation [5]. The polymer-protein conjugates enhance protein stability but reduce immunogenicity, whereas the polymer-drug conjugates display enhanced permeability and retention effects [6]. More recently, the polymeric nanoparticle poly-(lactic-co-glycolic acid) (PLGA) has also been used as a nanocarrier for drug delivery across the blood-brain barrier due to its biocompatibility and biodegradability, thereby ensuring safe therapy [7]. Inorganic ceramic nanoparticles such as silica, titania and alumina are also commonly being used for drug administration for cancer therapy due to their porous nature, although their applications are limited due to their non-biodegradable nature [8,9]. On the other hand, metallic nanoparticles, including superparamagnetic iron oxide nanoparticles, gold shell nanoparticles and titanium dioxide (TiO_2_) nanoparticles, are routinely used for magnetic resonance imaging contrast enhancement and as cancer drug carrier systems, whereas silver nanoparticles (AgNP) are being explored as antibacterial agents for treatment of infectious diseases, due to their ability to stabilize nanoparticles and favorable optical/chemical properties [10,11,12,13,14,15]. Notably, carbon nanoparticles, which are comprised of fullerenes and nanotubes, are the most widely used materials for drug delivery purposes due to the fact that fullerenes contain multiple attachment points responsible for tissue binding, and nanotubes offer high electrical conductivity and strength [16,17].

Nanoparticles have been used as a tool for the detection of disease biomarkers in both *in vivo* and *ex vivo* diagnostic applications, consequently leading to an advancement of proteomics and genomics technologies [18,19,20]. For example, streptadivin-coated fluorescent polystyrene nanospheres offer greater sensitivity in the detection of epidermal growth factor receptor (EGFR) in human carcinoma cells, thus providing a more sensitive tool for biomarker discovery [21]. Furthermore, an ultrasensitive nanoparticle-based assay for the detection of prostate-specific antigen (PSA) in the serum was developed, which can provide up to six orders of magnitude higher sensitivity than the conventional assay [22]. Therefore, nanoparticles have also gained popularity in the field of molecular diagnosis and imaging, due to their favorable physicochemical properties of small particle size, flexibility of surface coating and enhanced stability [23,24].

Nanotechnology has also found an application in molecular imaging, particularly in magnetic resonance imaging (MRI), fluorescence imaging, computed tomography imaging and ultrasound techniques [25,26,27]. Gadolinium-based paramagnetic nanoparticles targeting fibrin in atherosclerotic plaques allowed for more effective imaging as compared to the commonly used contrast agents; in turn promoting early detection of vulnerable plaques [28,29,30]. Moreover, nanoparticles have been shown to not only increase specificity of targeting but also increase/facilitate solubility, stability and absorption of the drug [31,32]. Particularly, nanoparticle formulations carrying anti-cancer drugs, including paclitaxel, 5-fluorouracil and doxorubicin, have been observed to be more efficient drug delivery systems, by enhancing the cytotoxic effects of the drug while reducing non-specific targeting of normal cells [33,34,35].

## 2. Toxicity of Nanoparticles

Despite the gaining popularity of nanotechnology in the field of medicine, their applications have been restricted due to their potential toxicity and long-term secondary adverse effects [2]. Nanotoxicology includes the study of the toxicity of nanomaterials to better understand and assess the health risks involved in the use of nanoparticles. The physicochemical properties of nanoparticles, such as small size, large surface area and flexible chemical composition/structure that favor their use in nanomedicine, have also been found to contribute to their enhanced toxicological side effects [36]. Specifically, particle size and surface area are considered important factors that contribute directly and significantly to toxicity of nanoparticles, with smaller sized nanoparticles exhibiting higher toxic effects due to increased surface area [37]. Apart from size, structure and shape of the nanoparticle also contribute to nanotoxicity. For example, studies with carbon nanofibers, single-wall nanotubes (SWCNTs) and multi-wall nanotubes (MWCNTs), have revealed that the toxicity of carbon material with high-aspect ratio is determined by particle form and dimensions [38]. Moreover, the nanoparticle surface dictates the adsorption of ions and biomolecules, thus influencing the cellular responses elicited, and thereby contributing to nanoparticle induced toxicity [39].

Humans can be exposed to nanomaterials via several routes such as inhalation, injection, oral ingestion and the dermal route. Specifically, the respiratory system, gastrointestinal tract, the circulatory system as well as the central nervous system are known to be adversely affected by nanoparticles [23]. *In vivo* experiments have revealed that carbon nanotubes are found to cause dose-dependent epithelioid granulomatous lesions in the lung and persistent interstitial inflammation on chronic exposure [40,41]. Furthermore, ceramic nanoparticles, commonly used for drug delivery, have been reported to exhibit oxidative stress/cytotoxic activity in the lungs, liver, heart, and brain, as well as have teratogenic/carcinogenic effects [42].

In addition to causing detrimental respiratory effects, nanoparticles administered via injection have been shown to enter the systemic circulation, causing secondary complications in the circulatory system and further gain access to the central nervous system. Engineered carbon nanoparticles and nanotubes were found to induce the aggregation of platelets *in vitro*, and thus enhance vascular thrombosis in rat carotid artery [43]. Furthermore, the effect of SWCNTs was studied in cellular models of human kidney and bronchi, where they were observed to induce cell apoptosis and decrease cell adhesion via either upregulating genes involved in cell death or downregulating genes associated with cell proliferation and survival [44,45]. Wistar rats injected intraperitoneally with 20 mg/kg titanium dioxide nanoparticles (TiO_2_NPs) every two days for 20 days, revealed an accumulation of TiO_2_NPs in the liver, lung and brain, and an increase in aspartate aminotransferase/alanine aminotransferase ratio (AST/ALT ratio), indicating subacute toxicity. In the injected rats, pathological changes were found in the liver and abnormal neuro-behavioural performance, as evidenced by the increased anxious index was observed, suggesting that TiO_2_NPs are able to translocate and biodistribute to various organs leading to toxicity effects [46].

Oral ingestion of a single dose of 500 mg/kg titanium dioxide (TiO_2_), zinc oxide (ZnO) and aluminium oxide (Al_2_O_3_) nanoparticles, were shown to result in nanoparticle translocation to the central nervous system. These nanoparticles accumulated in the brain and caused axillary toxicity, disrupting normal metabolism of neurotransmitters and ultimately leading to brain damage [47]. The effect of different sized TiO_2_ nanoparticles were studied in rat astrocytes, in which these nanoparticles were found to inhibit cell survival rates in a dose-dependent manner, with pathological effects such as blood-brain barrier destruction, cellular oedema and brain tissue necrosis [48]. Furthermore, nano-manganese dioxide (MnO_2_) was also found to cause dopaminergic neuronal dysfunction and astrocyte activation, thus affecting the learning abilities of rats [49].

Dermal exposure of nanoparticles is often mediated through the use of nanomaterial containing cosmetic products or wound dressings. For instance, sunscreens containing TiO_2_ were found to pass through the stratum corneum and in the deeper parts of hair follicles [50]. In addition, Acticoat, a nanocrystalline silver-coated wound dressing, is now being used for treatment in burn patients. Despite various studies reporting about the safety of Acticoat for the use on burn patients, silver toxicity was reported in a patient with 30% burns who had received the silver-coated dressing for treatment [51].

The accumulation of nanoparticles in various organs and adverse side effects have hindered their use in the field of nanomedicine, and have deterred full exploitation of their potential in molecular diagnostics and as drug delivery systems (Table 1). A better understanding of the mechanisms involved in nanotoxicity may provide clues for circumventing the toxicological effects of nanoparticles and may help to further develop/exploit nanoparticles in the field of nanomedicine.

**Table 1:** Overview of the different types of nanoparticles used in nanomedicine, and the toxicity associated.

Class/Type of Nanoparticles	Application in Nanomedicine	Toxicity
Polymeric nanoparticles		
Polysaccharide chitosan nanoparticles (CS-NPs)	Drug delivery [5]	Not reported
Poly-(lactic-co-glycolic acid) (PLGA)	Cancer therapy and drug delivery [7]	Not reported
Inorganic nanoparticles		
Silica nanoparticles	Drug delivery/Diagnostic imaging [9]	Platelet aggregation and physiological toxicity [52], reproductive toxicity [53]
Ceramic nanoparticles	Cancer drug delivery [8]	Oxidative stress/cytotoxic activity in the lungs, liver, heart, and brain [42]
Metallic nanoparticles		
Superparamagnetic iron oxide nanoparticles	Magnetic resonance imaging contrast enhancement, immunoassays and cancer drug carrier systems [11,12]	Oxidative stress and disturbance in iron homeostasis [54]
Gold shell nanoparticles	Biomedical imaging and therapeutics [13]	Hepatic and splenic toxicity [55]
Titanium dioxide	Cancer therapeutics [14]	Toxicity to the central nervous system [46,47]
Silver nanoparticles	Antibacterial agents [15]	ER stress response not only in the lung, liver and kidneys [56]
Carbon nanoparticles (fullerenes and nanotubes)	Drug delivery [16,17]	Pulmonary toxicity and interstitial inflammation [40,41]

## 3. Molecular Mechanisms Underlying Nanotoxicity

### 3.1. Oxidative Stress and DNA Damage

Nanoparticles are known to induce reactive oxygen species (ROS) production, leading to an oxidative stress when redox state of the cell is imbalanced [57,58,59,60]. ROS induction by nanoparticles is considered the primary cause of nanotoxicity, and has been attributed to the presence of pro-oxidant functional groups on their reactive surface or due to nanoparticle-cell interactions [61,62]. ROS production is a normal cellular process which is involved in varied aspects of cellular signaling, as well as in the defence mechanism of the immune system. However, in excess it has been found to cause severe damage to cellular macromolecules such as proteins, lipids and DNA, resulting in detrimental effects on cells.

*In vitro* studies with different sized (15, 30, 45 nm) cerium oxide nanoparticles indicated that they exert their toxicity through oxidative stress, which in turn brings about Nrf2-mediated induction of heme oxygenase-1 (HO-1) [63]. Furthermore, silver nanoparticles (AgNPs) of different sizes (4.7 and 42 nm) showed the induction of ROS, glutathione depletion, as well as a slight inhibition of superoxide dismutase [64]. Studies with gold nanoparticles (AuNPs) of sizes ranging from 5 to 250 nm have also revealed that smaller diameter nanoparticles with larger surface area produce higher amounts of ROS, thus establishing an inverse relationship between these two parameters [65]. Both *in vitro* and *in vivo* studies with silica nanoparticles indicated that single dose exposure to these nanoparticles leads to ROS induction, consequently activating pro-inflammatory responses [66]. ROS generation, decreased mitochondrial membrane potential, increased levels of lipid peroxide and decreased enzymatic activities of antioxidants were shown to be induced by single-walled carbon nanotubes [67]. Additionally, multi-walled carbon nanotubes also exhibited a dose-dependent induction of ROS [68].

Toxicity of nanoparticles is attributed to oxidative stress, followed by DNA damage and apoptosis. Nanoparticles can cause a wide variety of DNA damage, ranging from chromosomal fragmentation, DNA strand breakages and the induction of gene mutations [69,70,71,72]. AuNPs (20 nm size) at 1 nM concentration have been shown to exhibit DNA damage in the form of 8-hydroxydoxyguanosine (8OHdG) adducts formation in embryonic lung fibroblasts with a decreased expression of DNA repair and the cell cycle checkpoint genes *MAD2*, *cyclin B1* and *cyclin B2* [73]. Various studies have also confirmed the occurrence of DNA fragmentation and formation of oxidation-induced DNA adducts on exposure to metal oxide nanoparticles [74,75,76]. In response to this DNA damage, the cells either initiate DNA repair mechanisms or invoke cell cycle arrest and apoptosis. One of the major effector molecules activated in response to DNA damage is p53. It plays a central role in DNA repair and cell cycle arrest, thereby preventing mutagenic events favouring the process of carcinogenesis [77]. Cadmium-telluride quantum dots were found to significantly increase p53 levels and upregulate the p53-downstream effectors Bax, Puma and Noxa in human breast carcinoma cells [78]. Altered expression of DNA damage responsive genes has also been observed in response to nanoparticle exposure. Cultured human embryonic lung fibroblasts exposed to AuNPS showed the down-regulation of DNA repair genes *BRCA1*, *Hus1*, *ATLD/HNGS1* and *AT-V1/AT-V2* [73]. If the extent of DNA damage goes beyond the scope of repair by the DNA repair mechanisms of the body, the cells initiate a programmed cell death. Apoptosis is a highly complex and tightly regulated pathway involving several signaling molecules. Metal oxide nanoparticles including TiO_2_, ZnO, Fe_3_O_4_, Al_2_O_3_, and CrO_3_ of particle sizes ranging from 30 to 45 nm were found to induce apoptosis [79]. *In vivo* studies with the fruit fly *Drosophila* models showed that AgNP induces heat shock stress, oxidative stress, DNA damage and apoptosis, thereby mediating developmental and reproductive toxicity [80]. However, the exact cascade of signaling molecules mediating apoptosis in nanoparticle-induced toxicity is poorly studied [81], and thus attempts to better understand this mechanism may prove useful in reducing the toxicological side effects.

### 3.2. Inflammation-Mediated Nanotoxicity

Inflammation is a defence mechanism of the body that involves several immune regulatory molecules, following the infiltration of phagocytic cells. Several studies with single and multi-walled carbon nanotubes and fullerene derivatives have shown the induction of inflammation in varied cell types, including alveolar and bronchial epithelial cells, epidermal keratinocytes and cultured monocyte-macrophage cells [82,83,84,85]. More recently, a study was carried out to provide a mechanistic explanation for immune and inflammatory responses observed upon exposure to carbon nanoparticles. The computational model suggested that the carbon nanotubes and C_60_ fullerenes may be recognized as pathogens by the Toll-like receptors, triggering innate immune responses of the body and secretion of inflammatory protein mediators such as interleukins and chemokines [86]. Furthermore, activation of the complement cascade on exposure to liposomes and other lipid-based nanoparticles leads to hypersensitivity reactions and anaphylaxis [87,88,89]. However, the exact mechanism of how these complement proteins mediate nanotoxicity has not yet been elucidated [90]. Nanoparticle immunogenicity has also been attributed to their property of acting as adjuvants, thereby improving the antigenicity of conjugated weak antigens [91,92]. The ability of nanoparticles to serve as adjuvants is dependent on their size and surface charge, and dictates the type of cytokines that would be released [93]. Importantly, inflammation has been shown to directly cause toxicity and promote cell death through the induction of toxic by-products of inflammation such as ROS and complement proteins, as well as via receptor-induced apoptosis/necrosis [94]. These cascades have not been well explored in the context of nanoparticle-induced cytotoxicity, and investigations in this direction are required to fully identify and recognize the signaling networks mediating inflammation-driven cell death.

Interestingly, oxidative stress also results in the release of pro-inflammatory mediators through the principal cascades such as the NF-κB (Nuclear Factor-κB), mitogen-activated protein kinase (MAPK) and phosphoinositide 3-kinase (PI3-K) pathways [95,96], suggesting that oxidative stress is linked to inflammation reciprocally [81]. In the absence of a stimulus, NF-κB is sequestered in the cytoplasm by the Inhibitor of κB (IκB) family of inhibitors. However, in the event of oxidative stress, the IκB undergoes degradation, thus freeing NF-κB which then translocates into the nucleus to regulate the transcription of its target genes [97]. In support of this, the OH, HOCl, and ^1^O_2_ reactive species are known to induce the nuclear translocation and activation of NF-κB [98]. Both *in vitro* and *in vivo* studies showed that nanoparticle-induced lung injury and pulmonary fibrosis lead to the ROS-mediated activation of NF-κB and production of pro-inflammatory mediators such as TNF-α, IL-8, IL-2 and IL-6 [99,100]. Several metal oxide nanoparticles including zinc, cadmium, silica, and iron have also been shown to exert their toxicity via the production of inflammatory cytokines induced by NF-κB [101,102,103,104]. Furthermore, both single-walled and multi-walled carbon nanotubes were also shown to promote inflammatory responses in mice with the production of TNF-α and Monocyte Chemoattractant Protein-1 (MCP-1) [105].

The MAPK pathway regulates a diverse range of cellular responses, including cell proliferation, differentiation, mitosis, cell survival and apoptosis. They are a family of serine/threonine protein kinases that include growth factor-regulated extracellular signal-related kinases (ERK) and the stress-activated MAPK, c-Jun NH2-terminal kinases (JNK) and p38 MAPK. The ERKs are mainly associated with cell proliferation and differentiation, whereas the JNKs and p38 MAPKs are known to regulate responses to cellular stresses [106]. IL-8 production via the p38 MAPK and/or ERK pathway was shown to mediate toxicity in human bronchial epithelial cell line upon treatment with titanium dioxide nanoparticles [107]. *In vivo* nanotoxicity studies with the model organism *C. elegans* to assess the effects of AgNPs (size ranging from 20 to 30 nm), showed that the toxicity was mediated by increased ROS formation, followed by the increased expression of PMK-1 p38 MAPK and hypoxia-inducible factor (HIF-1) [108]. Moreover, the toxicity of silica nanoparticles which hinders their application as drug delivery systems has been attributed to the activation of JNK, p53 and NF-κB pathways and an elevated expression of pro-inflammatory factors IL-6, IL-8 and MCP-1 [109]. Besides, single-walled carbon nanotubes (0.8–2 nm) were also shown to cause potential adverse cellular responses in mesothelial cells via the activation of signaling molecules, including ARP, AP-1, NF-κB, p38 and Akt, in a dose-dependent manner [110]. On the other hand, the PI3-K/Akt/mTOR pathway is one of the principal signaling cascades regulating cell cycle, thus making it critical for cell survival and growth. PI3-K signaling was found to cause an overexpression of Cox-2, iNOS and pro-inflammatory cytokines (IL-6, IFN-γ, TNF-α, IL-17 and regulatory cytokine IL-10) in macrophages upon exposure to zinc oxide nanoparticles [111]. Furthermore, silica nanoparticles (average diameter of 62.1 ± 7.2 nm) were shown to induce inflammatory responses and activate autophagy via the PI3-K/Akt/mTOR pathway [112].

## 4. Possible Strategies to Circumvent Nanotoxicity

Oxidative stress results from imbalances in the redox state of the cell. The redox state is disturbed by ROS production in response to nanoparticle exposure and/or nanoparticle-induced inflammation cascade. Many *in vitro* and *in vivo* studies have recognized the central role of oxidative stress in mediating nanotoxicity and therefore, the approach of preventing oxidative stress is an ideal strategy in circumventing it.

One possible way to prevent oxidative stress-mediated nanotoxicity is the introduction of ascorbic acid upon nanoparticle exposure. Ascorbic acid, also known as vitamin C, is an antioxidant capable of scavenging free radicals [113]. By introducing ascorbic acid into AgNP-treated acute myeloid leukemia cells there is a complete decrease in ROS production in the cells. Concomitantly, ascorbic acid also led to a decrease in AgNP-induced mitochondria damage, apoptosis and DNA damage, reducing the toxic effects induced by AgNPs [114]. Similar mitigation of ROS generation and glutathione depletion were also observed when ascorbic acid was added to human lung epithelial (A549) cells treated with nickel ferrite nanoparticles (26 nm in diameter) [115]. An *in vivo Drosophila melanogaster* study has also shown a decrease in nanotoxicity when ascorbic acid was supplemented in the diet of *Drosophila* exposed to AgNPs [116]. *In vivo* studies with rats have further revealed that acute oxidative stress and inflammation induced by ZnO nanoparticles (of particle size 21 nm) were alleviated when 1% aqueous ascorbic acid was given as drinking water [117]. Hence, the administration of ascorbic acid after nanomaterial exposure is a feasible strategy to overcome nanotoxicity, both *in vitro* and *in vitro*.

Quercetin, a naturally occurring flavonoid in many plants and food, is an anti-oxidant having free radical scavenging ability. Quercetin has been found to reduce Fe_2_O_3_ nanoparticles-induced oxidative injury and inflammation by increasing Bad phosphorylation and Nrf2 translocation through PI3-K/Akt dependent pathways [118]. *In vivo* studies have also revealed that TiO_2_ NPs induced liver and kidney oxidative stress can be circumvented by treatment with quercetin [119].

Surface modification of nanoparticles can also be carried out to decrease nanotoxicity. An example would be the encapsulation of ascorbic acid with poly (l-glutamic acid)-capped silver nanoparticles (AgNpPGA) within a poly (lactide-co-glycolide) (PLGA) polymeric matrix (PLGA/AgNpPGA/ascorbic acid particles). A reduction in ROS generation was observed in HepG2 cells treated with the PLGA/AgNpPGA/ascorbic acid particles as compared to control cells, suggesting that nanoparticles encapsulated with ascorbic acid can reduce oxidative stress in cells, possibly decreasing the nanotoxic effects of nanoparticles [120]. Copper nanoparticles coated with polysaccharides such as chitosan have also shown to decrease *in vitro* toxicity and ROS generation, although the modification increased inflammatory responses when administered via the lung [121]. Furthermore, Fe_2_O_3_ nanoparticles coated with chitosan resulted in a decrease in cellular damage and moderated ROS production, thereby, reducing the cytotoxic effects of the nanoparticles [122]. Polymer coatings such as polyethylene glycol (PEG) on superparamagnetic iron oxide nanoparticles (SPIONs) have also effectively reduced nanoparticle cytotoxicity by reducing ROS formation. The PEG coating blocks ROS interaction with the Fe_2_O_3_ nanoparticles (of diameter 5 and 30 nm), thus preventing formation of hydroxyl radicals and allowing the cell’s antioxidant defense mechanisms to neutralize ROS before they become toxic [123].

The NF-κB, MAPK and PI3-K pathways facilitate nanoparticle-induced inflammation, and release of several pro-inflammatory cytokines and chemokines including TNF-α, IL-6 and IL-8, leading to cytotoxicity and cell death. With the intention of combating inflammation, the Jun/AP-1 pathway components have been modulated providing new avenues for therapeutic interventions [124]. Hence, targeting these signaling molecules also holds promise as an effective tool to evade/circumvent the inflammation-mediated toxicity, thereby allowing for the development of nanoparticle-based applications in the field of medicine.

## 5. Conclusions

**Figure 1:**
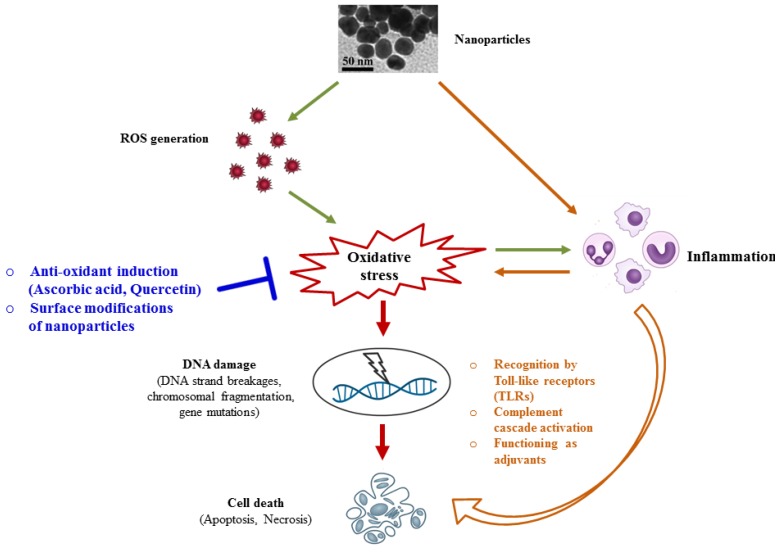
Overview of the signaling cascades mediating nanotoxicity, and possible strategies to circumvent the toxicity.

Nanoparticles hold great potential in the field of nanomedicine due to their favourable physicochemical properties. However, nanotoxicity has become a growing concern of nanotechnology. Numerous *in vitro* and *in vivo* studies have consistently demonstrated that nanoparticles induce ROS production, causing an imbalance in the redox state and subsequently leading to an oxidative stress in the cell, as illustrated in Figure 1. Therefore, strategies targeting the oxidative stress hold great potential in further developing nanoparticle-based tools for medical applications by eradicating nanotoxicity.
